# Supplementary material for: Selenium and Vitamin E Concentrations in Miranda Jennies and Foals (Equus asinus) in Northeast Portugal
Source: Animals (Basel). 2021 Jun 14;11(6):1772. doi: 10.3390/ani11061772 (PMC8231900; doi:10.3390/ani11061772)
Supplement: Supplementary file 1 [file animals-11-01772-s001.zip › animals-1201341-supplementary.pdf]

Supplementary Table S1. Bayesian inference for One-Way ANOVA to test for differences in the posterior mean for vitamin E (mg/L) and selenium (µg/L) in jennies and foals between sampling locations in Miranda Donkeys.

	**Jenny vitamin E (mg/L)**		**Jenny selenium (µg/L)**		**Foal vitamin E (mg/L)**		**Foal selenium (µg/L)**	
**Groups**	**Between Groups**	**Within Groups**	**Between Groups**	**Within Groups**	**Between Groups**	**Within Groups**	**Between Groups**	**Within Groups**
Sum of Squares	0.748	84.001	8215.446	14314.242	6.393	343.293	1280.261	12040.247
df	1	31	1	31	1	31	1	31
Mean Square	0.748	2.71	8215.446	461.75	6.393	11.074	1280.261	388.395
F	0.276		17.792		0.577		3.296	
p-value	0.603		0.000		0.453		0.079	
Bayes Factor	0.154		128.317		0.179		0.624	
**Categories**	**Paradela**	**Atenor**	**Paradela**	**Atenor**	**Paradela**	**Atenor**	**Paradela**	**Atenor**
Posterior Mode	4.651	4.964	9.612	42.412	4.022	3.107	6.43	19.378
Posterior Mean	4.651	4.964	9.612	42.412	4.022	3.107	6.43	19.378
Posterior Variance	0.241	0.138	41.133	23.505	0.986	0.564	34.598	19.771
95% CI Lower Bound	3.682	4.231	−3.039	32.849	2.062	1.626	−5.173	10.607
95% CI Upper Bound	5.62	5.696	22.264	51.976	5.981	4.588	18.033	28.149

df: degrees of freedom; F: Snedecor's F; CI: Confidence interval.

Supplementary Table S2. Bayesian inference for One-Way ANOVA to test for differences in the mean for vitamin E (mg/L) and selenium (μg/L) between genders in Miranda jennies and foals.

	**Jenny vitamin E (mg/L)**		**Jenny selenium (μg/L)**		**Foal vitamin E (mg/L)**		**Foal selenium (μg/L)**	
**Groups**	**Between Groups**	**Within Groups**	**Between Groups**	**Within Groups**	**Between Groups**	**Within Groups**	**Between Groups**	**Within Groups**
Sum of Squares	1.078	83.672	2237.325	20292.363	86.749	262.937	1578.133	11742.374
df	1	31	1	31	1	31	1	31
Mean Square	1.078	2.699	2237.325	654.592	86.749	8.482	1578.133	378.786
F	0.399		3.418		10.228		4.166	
Sig.	0.532		0.074		0.003		0.050	
Bayes Factor	0.164		0.658		10.086		0.911	
**Categories**	**Male**	**Female**	**Male**	**Female**	**Male**	**Female**	**Male**	**Female**
Posterior Mean/Mode	5.233	4.765	13.018	34.367	0	4.204	0	17.93
Posterior Variance	0.481	0.107	116.623	25.916	1.511	0.336	67.485	14.997
95% CI Lower Bound	3.865	4.12	−8.284	24.324	−2.425	3.061	-16.205	10.291
95% CI Upper Bound	6.601	5.41	34.321	44.409	2.425	5.347	16.205	25.569

df: degrees of freedom; F: Snedecor's F; CI: Confidence interval.

Supplementary Table S3. Bayesian inference for One-Way ANOVA to test for differences in the posterior mean for vitamin E (mg/ L) and selenium (µg/L) across Body Score Condition (BCS) categories in Miranda jennies and foals.

Factor	Variable	Groups	Sum of Squares	df	Mean Square	F	Sig.	Bayes Factor
BCS	Jenny vitamin E (mg/L)	Between Groups	18.78	4	4.695	1.993	0.123	0.072
		Within Groups	65.97	28	2.356			
	Jenny selenium (µg/L)	Between Groups	3944.673	4	986.168	1.486	0.233	0.032
		Within Groups	18585.015	28	663.751			
	Foal vitamin E (mg/L)	Between Groups	46.832	4	11.708	1.082	0.384	0.016
		Within Groups	302.855	28	10.816			
	Foal selenium (µg/L)	Between Groups	956.508	4	239.127	0.542	0.707	0.006
		Within Groups	12363.999	28	441.571			

Variable	BCS	Posterior Mean/Mode	Posterior Variance	95%CI Lower Bound	95%CI Upper Bound
Jenny vitamin E (mg/L)	2	4.855	0.317	3.743	5.967
	2.5	5.897	0.282	4.849	6.945
	3	4.564	0.231	3.616	5.512
	3.5	3.46	1.269	1.237	5.683
	4	3.673	0.846	1.858	5.489
Jenny selenium (µg/L)	2	21.381	89.351	2.723	40.04
	2.5	28.457	79.423	10.865	46.048
	3	39.937	64.983	24.025	55.849
	3.5	53.765	357.404	16.448	91.082
	4	10.67	238.269	−19.799	41.139
Foal vitamin E (mg/L)	2	2.391	1.456	0.009	4.773
	2.5	3.8	1.294	1.554	6.046
	3	2.835	1.059	0.804	4.867
	3.5	4.56	5.824	−0.204	9.324
	4	6.62	3.883	2.731	10.509
Foal selenium (µg/L)	2	15.383	59.442	0.164	30.601
	2.5	22.552	52.838	8.204	36.9
	3	10.249	43.231	−2.729	23.227
	3.5	6.54	237.769	−23.897	36.977
	4	10.75	158.513	−14.102	35.602

df: degrees of freedom; F: Snedecor's F; CI: Confidence interval.

Supplementary Table S4. Bayesian inference for Pearson's linear correlation between age of jennies' levels of selenium (µg/L) and vitamin E (mg/L) in Miranda jennies and foals.

Parameter	Jenny vitamin E (mg/L)	Jenny selenium (µg/L)	Foal vitamin E (mg/L)	Foal selenium (µg/L)
Mode	−0.261	0.189	0.375	0.330
Mean	−0.240	0.174	0.346	0.304
Variance	0.025	0.026	0.022	0.023
95% CI Lower Bound	−0.542	−0.144	0.052	0.001
95% CI Upper Bound	0.070	0.484	0.624	0.592

CI: Confidence interval.

Supplementary Table S5. Bayesian inference for One-Way ANOVA to test for differences in the posterior mean for vitamin E (mg/L) and selenium (µg/L) and across diet categories in Miranda jennies and foals.

Factor	Variable	Groups	Sum of Squares	df	Mean Square	F	Sig.	Bayes Factor
Diet	Jenny vitamin E (mg/L)	Between Groups	11.874	2.000	5.937	2.444	0.104	0.259
		Within Groups	72.875	30.000	2.429			
	Jenny selenium (µg/L)	Between Groups	7560.248	2.000	3780.124	7.576	0.002	11.512
		Within Groups	14969.440	30.000	498.981			
	Foal vitamin E (mg/L)	Between Groups	178.647	2.000	89.323	15.667	0.000	1024.893
		Within Groups	171.039	30.000	5.701			
	Foal selenium (µg/L)	Between Groups	3111.042	2.000	1555.521	4.571	0.019	1.410
		Within Groups	10209.465	30.000	340.316			

Variable	Forrage	Posterior Mean/Mode	Posterior Variance	95% CI Lower Bound	95% CI Upper Bound
Jenny vitamin E (mg/L)	Oat (straw with grain)	4.336	0.137	3.606	5.066
	Oat (straw with grain) and hay	5.473	0.651	3.881	7.064
	Other	5.578	0.260	4.571	6.585
Jenny selenium (µg/L)	Oat (straw with grain)	34.571	28.138	24.105	45.036
	Oat (straw with grain) and hay	59.820	133.656	37.010	82.630
	Other	10.989	53.462	−3.437	25.415
Foal vitamin E (mg/L)	Oat (straw with grain)	5.221	0.322	4.102	6.339
	Oat (straw with grain) and hay	3.578	1.527	1.139	6.016
	Other	0.000	0.611	−1.542	1.542
Foal selenium (µg/L)	Oat (straw with grain)	21.511	19.191	12.867	30.154
	Oat (straw with grain) and hay	18.850	91.156	0.012	37.688
	Other	0.000	36.462	−11.914	11.914

df: degrees of freedom; F: Snedecor's F; CI: Confidence interval.

Supplementary Table S6. Bayesian inference for One-Way ANOVA to test for differences in the posterior mean for vitamin E (mg/L) and selenium (µg/L) across supplements categories in Miranda jennies and foals.

Factor	Variable	Groups	Sum of Squares	df	Mean Square	F	Sig.	Bayes Factor
Supplements	Jenny vitamin E (mg/ L)	Between Groups	2.228	2.000	1.114	0.405	0.671	0.041
		Within Groups	82.521	30.000	2.751			
	Jenny selenium (µg/L)	Between Groups	15201.376	2.000	7600.688	31.115	0.000	395935.603
		Within Groups	7328.311	30.000	244.277			
	Foal vitamin E (mg/L)	Between Groups	24.152	2.000	12.076	1.113	0.342	0.080
		Within Groups	325.534	30.000	10.851			
	Foal selenium (µg/L)	Between Groups	4553.885	2.000	2276.943	7.792	0.002	13.241
		Within Groups	8766.622	30.000	292.221			

Variable	Supplements	Posterior Mean/Mode	Posterior Variance	95% CI Lower Bound	95% CI Upper Bound
Jenny vitamin E (mg/L)	No	4.859	0.164	4.061	5.657
	Salt blocs ad libitum	5.384	0.589	3.869	6.899
	Extrafeed	4.567	0.295	3.496	5.638
Jenny selenium (µg/L)	No	10.893	14.540	3.369	18.416
	Salt blocs ad libitum	53.744	52.345	39.469	68.019
	Extrafeed	54.122	26.173	44.028	64.216
Foal vitamin E (mg/L)	No	2.681	0.646	1.095	4.267
	Salt blocs ad libitum	3.920	2.325	0.911	6.929
	Extrafeed	4.564	1.163	2.437	6.691
Foal selenium (µg/L)	No	4.287	17.394	−3.942	12.515
	Salt blocs ad libitum	20.968	62.619	5.355	36.581
	Extrafeed	30.210	31.309	19.170	41.250

df: degrees of freedom; F: Snedecor's F; CI: Confidence interval.

Supplementary Table S7. Bayesian inference for One-Way ANOVA to test for differences in the posterior mean vitamin E (mg/ L) and selenium (µg/L) across foal death or conditions categories in Miranda Donkeys.

Factor	Variable	Groups	Sum of Squares	df	Mean Square	F	Sig.	Bayes Factor
Foal death or conditions	Jenny vitamin E (mg/ L)	Between Groups	49.439	3.000	16.480	13.535	0.001	1959.551
		Within Groups	35.310	29.000	1.218			
	Jenny selenium (µg/L)	Between Groups	4109.974	3.000	1369.991	2.157	0.115	0.130
		Within Groups	18419.714	29.000	635.163			
	Foal vitamin E (mg/ L)	Between Groups	61.029	3.000	20.343	2.044	0.130	0.114
		Within Groups	288.657	29.000	9.954			
	Foal selenium (µg/L)	Between Groups	1217.408	3.000	405.803	0.972	0.419	0.029
		Within Groups	12103.099	29.000	417.348			

Variable	Foal death or conditions	Posterior Mean/Mode	Posterior Variance	95% CI Lower Bound	95% CI Upper Bound
Jenny vitamin E (mg/L)	Does not apply	4.646	0.048	4.212	5.080
	Foal found dead after non assisted parturation	5.307	0.436	4.004	6.610
	Weak foal but independent. There was a twin mumified foetus in the uterus associated with endometritis.	3.585	0.654	1.989	5.181
	Foal born with carpal flexural deformity	11.520	1.308	9.263	13.777
Jenny selenium (µg/L)	Does not apply	29.463	25.267	19.543	39.383
	Foal found dead after non assisted parturation	60.737	227.404	30.977	90.496
	Weak foal but independent. There was a twin mumified foetus in the uterus associated with endometritis.	10.495	341.106	−25.953	46.943
	Foal born with carpal flexural deformity	7.310	682.212	−44.235	58.855
Foal vitamin E (mg/L)	Does not apply	4.049	0.396	2.807	5.291
	Foal found dead after non assisted parturation	0.000	3.564	−3.725	3.725
	Weak foal but independent. There was a twin mumified foetus in the uterus associated with endometritis.	2.085	5.346	−2.478	6.648
	Foal born with carpal flexural deformity	0.000	10.691	−6.453	6.453
Foal selenium (µg/L)	Does not apply	16.602	9.432	25.514	6.453
	Foal found dead after non assisted parturation	0.000	149.421	−24.123	24.123
	Weak foal but independent. There was a twin mumified foetus in the uterus associated with endometritis.	6.170	224.131	−23.374	35.714
	Foal born with carpal flexural deformity	0.000	448.263	-41.782	41.782

df: degrees of freedom; F: Snedecor's F; CI: Confidence interval.

Supplementary Table S8. Bayesian inference for One-Way ANOVA to test for differences in the posterior mean for vitamin E (mg/L) and selenium (µg/L) in Miranda jennies and foals across number of previous parturitions in Miranda Donkeys.

	Variable	Groups	Sum of Squares	df	Mean Square	F	Sig.	Bayes Factor
Number of previous parturitions	Jenny vitamin E (mg/L)	Between Groups	6.099	3.000	2.033	0.750	0.531	0.021
		Within Groups	78.650	29.000	2.712			
	Jenny selenium (µg/L)	Between Groups	15384.248	3.000	5128.083	20.812	0.000	93165.797
		Within Groups	7145.439	29.000	246.394			
	Foal vitamin E (mg/L)	Between Groups	27.410	3.000	9.137	0.822	0.492	0.023
		Within Groups	322.277	29.000	11.113			
	Foal selenium (µg/L)	Between Groups	909.067	3.000	303.022	0.708	0.555	0.020
		Within Groups	12411.440	29.000	427.981			

Variable	Number of previous parturitions	Posterior Mean/Mode	Posterior Variance	95% CI Lower Bound	95% CI Upper Bound
Jenny vitamin E (mg/L)	0	4.843	0.146	4.090	5.596
	1	5.170	0.728	3.486	6.854
	2	5.143	0.416	3.870	6.416
	8	3.255	1.456	0.873	5.637
Jenny selenium (µg/L)	0	17.292	13.232	10.113	24.470
	1	78.960	66.161	62.908	95.012
	2	46.147	37.807	34.013	58.281
	8	10.655	132.323	−12.046	33.356
Foal vitamin E (mg/L)	0	2.897	0.597	1.372	4.422
	1	5.700	2.984	2.291	9.109
	2	3.489	1.705	0.912	6.066
	8	4.170	5.968	−0.651	8.991
Foal selenium (µg/L)	0	10.833	22.984	1.372	20.294
	1	22.565	114.921	1.409	43.721
	2	21.786	65.669	5.794	37.778
	8	12.340	229.841	−17.578	42.258

df: degrees of freedom; F: Snedecor's F; CI: Confidence interval.

Supplementary Table S9. Bayesian inference for One-Way ANOVA to test for differences in the posterior mean for vitamin E (mg/L) and selenium (μg/L) in jennies and foals across month of breeding in Miranda Donkeys.

Factor	Variable	Groups	Sum of Squares	df	Mean Square	F	Sig.	Bayes Factor
Month of breeding	Jenny vitamin E (mg/L)	Between Groups	19.903	6.000	3.317	1.330	0.280	0.009
		Within Groups	64.846	26.000	2.494			
	Jenny selenium (μg/L)	Between Groups	9762.264	6.000	1627.044	3.313	0.015	0.490
		Within Groups	12767.423	26.000	491.055			
	Foal vitamin E (mg/L)	Between Groups	183.417	6.000	30.569	4.780	0.002	4.906
		Within Groups	166.269	26.000	6.395			
	Foal selenium (μg/L)	Between Groups	9074.844	6.000	1512.474	9.262	0.000	882.299
		Within Groups	4245.663	26.000	163.295			

Variable	month of breeding	Posterior Mean/Mode	Posterior Variance	95% CI Lower Bound	95% CI Upper Bound
Jenny vitamin E (mg/L)	Missing	5.996	0.386	4.769	7.223
	January	4.603	0.901	2.729	6.478
	March	3.255	1.351	0.960	5.550
	April	4.713	0.208	3.813	5.613
	May	5.113	0.675	3.489	6.736
	July	3.285	1.351	0.990	5.580
	October	4.735	1.351	2.440	7.030
Jenny selenium (μg/L)	Missing	12.209	75.997	−5.008	29.425
	January	8.143	177.325	−18.155	34.442
	March	10.655	265.988	−21.554	42.864
	April	43.319	40.921	30.686	55.953
	May	54.225	132.994	31.450	77
	July	11.860	265.988	−20.349	44.069
	October	35.520	265.988	3.311	67.729
Foal vitamin E (mg/ L)	Missing	0.000	0.990	−1.965	1.965
	January	0.000	2.309	−3.001	3.001
	March	4.170	3.464	0.494	7.846
	April	4.651	0.533	3.209	6.092
	May	5.670	1.732	3.071	8.269
	July	4.090	3.464	0.414	7.766
	October	6.920	3.464	3.244	10.596
Foal selenium (μg/L)	Missing	0.000	25.272	−9.928	9.928
	January	0.000	58.968	−15.165	15.165
	March	12.340	88.451	−6.234	30.914
	April	11.655	13.608	4.370	18.941
	May	48.850	44.226	35.717	61.983
	July	9.440	88.451	−9.134	28.014
	October	46.810	88.451	28.236	65.384

df: degrees of freedom; F: Snedecor's F; CI: Confidence interval.
